# Increased pathogenicity and transmission of SARS-CoV-2 Omicron XBB.1.9 subvariants, including HK.3 and EG.5.1, relative to BA.2

**DOI:** 10.1128/jvi.01342-25

**Published:** 2025-11-18

**Authors:** Qiushi Jin, Ruixue Liu, Wenqi Wang, Jichen Xie, Fang Yan, Tiecheng Wang, Haiyang Xiang, Xianzhu Xia, Jianmin Li, Xuefeng Wang, Yuwei Gao

**Affiliations:** 1 Changchun Veterinary Research Institute, Chinese Academy of Agricultural Sciences 12661, Changchun, China; 2 College of Veterinary Medicine, Northeast Agricultural University 12430, Harbin, China; 3 College of Veterinary Medicine, Shanxi Agricultural University 74600, Jinzhong, China; 4 College of life sciences, Northeast Normal University 47821, Changchun, China; 5 State Key Laboratory of Reproductive Medicine and Offspring Health, Jiangsu Laboratory Animal Center, Jiangsu Animal Experimental Center of Medicine and Pharmacy, Department of Cell Biology, Animal Core facility, Key Laboratory of Model Animal, Collaborative Innovation Center for Cardiovascular Disease Translational Medicine, National Vaccine Innovation Platform, Nanjing Medical University 12461, Nanjing, China; 6 College of Wildlife and Nature Reserves, Northeast Forestry University 47820, Harbin, China; 7 Jiangsu Co-innovation Center for Prevention and Control of Important Animal Infectious Diseases and Zoonoses, Yangzhou University 38043, Yangzhou, China; University of Michigan Medical School, Ann Arbor, Michigan, USA

**Keywords:** SARS-CoV-2, Omicron, HK.3, XBB.1.9, transmission

## Abstract

With the SARS-CoV-2 Omicron XBB.1.9 subvariants circulating worldwide, two XBB.1.9 variants, EG.5.1 and HK.3, spread rapidly and became dominant in mid-2023. However, the spike features, pathogenicity, and transmissibility of HK.3 are largely unknown. Here, we performed multiscale investigations to reveal the virological features of XBB.1.9 subvariants, including the newly emerging HK.3. HK.3 revealed high replication efficiency and enhanced TMPRSS2 utilization *in vitro*. The HK.3 spike exhibited enhanced processing, although its infectivity, fusogenicity, and human ACE2 (hACE2) binding affinity were comparable to those of the EG.5 and XBB.1 spikes. All XBB.1.9.1, EG.5.1, and HK.3 strains demonstrated efficient transmission in hamsters, although XBB.1.9.1 exhibited stronger fitness in the upper airways. XBB.1.9.1, EG.5.1, and HK.3 exhibited greater pathogenicity than BA.2 in H11-K18-hACE2 hamsters. Our studies provide insights into the newly emerging pathogens EG.5.1 and HK.3.

**IMPORTANCE:**

SARS-CoV-2 Omicron continues to circulate and evolve into novel lineages with indistinguishable pathogenicity and transmission. Ancestral Omicron lineages, such as BA.1 and BA.2, revealed attenuated pathogenicity and transmission, at least in animal models. However, on a previously reported Omicron-sensitive H11-K18-hACE2 hamster model, the infections of XBB.1.9 lineages, EG.5, and HK.3 led to faster lethality and more severe terminal bronchioles symptom than BA.2. They also revealed efficient transmission in a hamster model, which corresponds well with their prevalence in multiple countries. Our study highlights the importance of surveillance and virological studies on epidemic Omicron subvariants.

## INTRODUCTION

The coronavirus disease 2019 (COVID-19) pandemic still lingers globally. One of the greatest challenges during the COVID-19 pandemic was the speed at which the causative agent severe acute respiratory syndrome coronavirus 2 (SARS-CoV-2) mutated. The Omicron BA.1 variant emerged in November 2021 and was characterized by more than 30 new mutations in the spike alone, and subsequent Omicron sublineages have continued to accumulate additional mutations (1). Identified in September 2022, the XBB lineage originated from a recombination of two BA.2-derived variants (BJ.1 and BM.1.1.1), mainly includes XBB.1.5, XBB.1.9, XBB.1.16, and XBB.2.3, and has progressively replaced most of the previous Omicron strains. These variants exhibit notable changes in virological characteristics, including increased transmissibility and obvious immune evasion (2–5).

EG.5.1, a subvariant representing most EG.5 strains (XBB.1.9.2.5), quickly spread in several areas of the world and replaced the previous XBB.1.5, XBB.1.9, and XBB.1.16 variants, which also evade most XBB.1.5-neutralizing antibodies (6). EG.5.1 has further evolved into a descendant lineage bearing the spike L455F mutation and has been named HK.3 (XBB.1.9.2.5.1.1.3), which showed increased transmission (7).

In this work, we investigated the *in vitro* and *in vivo* virological characteristics of isolates of XBB.1.9 subvariants. The *in vitro* replication kinetics of XBB.1.9.1, EG.5.1, and HK.3 were compared with those of the former epidemic strain BA.2. The pathogenicity of XBB.1.9.1, EG.5.1, and HK.3 in wild-type and K18-hACE2 rodents was determined. We also demonstrated the airborne transmission of XBB.1.9.1, EG.5.1, and HK.3 in hamsters. Moreover, as the spike protein universally impacts the infection and pathogenicity of SARS-CoV-2 via its functional characteristics (8–11), changes in spike features were also studied.

## RESULTS

### Prevalence and mutations of XBB.1.9 subvariants

**Fig 1:**
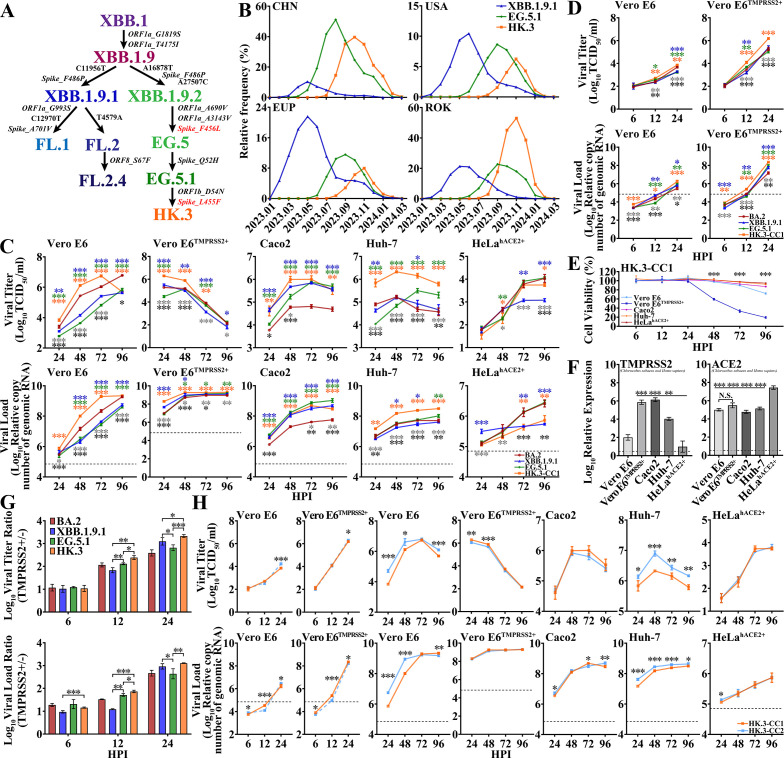
Evolution, prevalence, and replicative kinetics of XBB.1.9 subvariants. (**A**) Evolutionary origins of the XBB.1.9 sublineages, including XBB.1.9.1, EG.5.1, and HK.3. Synonymous mutations in nucleotides and amino acid mutations are shown in bold and bold-italic font, respectively. (**B**) Prevalence of XBB.1.9.1 (blue), EG.5.1 (green), and HK.3 (orange) in China (CHN), the United States (USA), Europe (EUP), and the Republic of Korea (ROK) for 14 months from January 2023 (2023.01) to March 2024 (2024.03). (**C and D**). Replicative kinetics of BA.2 (dark red), XBB.1.9.1 (blue), EG.5.1 (green), and HK.3 (orange) in terms of viral titers (upper panel) and viral loads (lower panel) in Vero E6, Vero E6^TMPRSS2+^, HeLa^hACE2+^, Huh-7, and Caco2 cells. Cells were infected at an MOI of 0.01. The significance of the differences in replication between BA.2 and XBB.1.9.1, EG.5.1, or HK.3 is indicated above the lines by the asterisks in colors corresponding to the individual viruses. The significance of the differences in replication between HK.3 and XBB.1.9.1 or EG.5.1 is indicated by gray or black asterisks below the lines. A detection reference (from a weakly positive sample, CT = 27.0) is represented by dashed lines. (**E**) Viability of HK.3-infected cells. Significance of viability differences between Vero E6 and Vero E6^TMPRSS2+^ cells is revealed. (**F**) Relative RNA expressions of TMPRSS2 (left) and ACE2 (right). Significance of the differences in TMPRSS2 expression between Vero E6 and other cells and in ACE2 expression between HeLa^hACE2+^ cells and others is indicated. (**G**) Ratio of viral titers (upper panel) and viral loads (lower panel) in Vero E6 cells with high versus low TMPRSS2 expression. (**H**) Replicative kinetics of two HK.3 isolates. Statistical analyses were conducted using Student’s *t*-test. Significances: *P* < 0.05 (*), *P* < 0.01 (**), or *P* < 0.001 (***). Viral titer reflects the number of infectious viral particles (TCID_50_/mL), whereas viral load represents RNA replication levels (copy number of genomic RNA).

Compared to XBB.1, XBB.1.9 bears the amino acid mutations G1819S and T4175I in ORF1a (Fig. 1A), with two diverging sublineages, both bearing the S486P mutation in the spike protein. One cluster was XBB.1.9.1 with the spike S486P and the nonsense C11956T mutations. The other cluster was XBB.1.9.2, which has the spike S486P mutation and the nonsense T23018C and A27507C mutations. The geographic distribution of XBB.1.9.1 and XBB.1.9.2 mainly includes Indonesia, England, the United States, and China. As a subvariant of XBB.1.9.2, EG.5.1 bears the spike Q52H/F456L double mutations, and its subvariant HK.3 contains a further L455F mutation in the spike protein. XBB.1.9.1 was one of the fastest growing variants in several areas of the world, including China, the United States, Europe, South Korea, and Singapore, starting in early January 2023; however, this variant was quickly replaced by XBB.1.9.2 within two to three months (Fig. 1B). Since May 2023, the prevalence of EG.5.1 and HK.3 has continued to increase in China after their emergence, accounting for up to 50% and 40% of new infections, respectively. Moreover, from July 2023 to February 2024, the relative prevalence of HK.3 reached 8%, 7%, and 52%, respectively, in the United States, Europe, and South Korea. XBB.1.9 variants exhibit notably increased transmissibility, and their remarkable immune evasion also triggered concern (7, 12, 13). Thus, we subsequently investigated the virological characteristics of XBB.1.9.1, EG.5.1, and HK.3.

### *In vitro* replication of Omicron XBB.1.9 subvariants

The replication kinetics of BA.2, XBB.1.9.1, and the subvariants EG.5.1 and HK.3 were compared by determining viral loads and viral titers from 24 to 96 h post-infection (HPI) in Vero E6, Vero E6^TMPRSS2+^, Caco2, Huh-7, and HeLa^hACE2+^ cells (Fig. 1C). The replication efficiency of HK.3 demonstrated superiority over BA.2, EG.5.1, and XBB.1.9.1 in both Vero E6 and Vero E6^TMPRSS2+^ cells. In Vero E6 cells, HK.3 demonstrated accelerated replication kinetics, of which the viral titer reached a peak value of 5.6 × 10^6^ TCID_50_/mL at 72 HPI. In contrast, replication peaks of XBB.1.9.1 and EG.5.1 did not emerge until 96 HPI, with lower viral titers of 4.4 × 10^5^ TCID_50_/mL and 7.1 × 10^5^ TCID_50_/mL, respectively. As early as 24 HPI, viral titers of HK.3 became 6.2- and 63.8-fold higher than those of XBB.1.9.1 and EG.5.1 in Vero E6^TMPRSS2+^ cells. Similarly, replication of HK.3 slightly or far exceeded other Omicron variants in Caco2 and Huh-7 cells, respectively. Nevertheless, propagation of all Omicron variants exhibited low efficiency in HeLa^hACE2+^ cells, in which most infectious viruses barely reached about 10^4^ TCID_50_/mL until 72 to 96 HPI. Results of viral RNA loads corroborate these findings. Infectious viruses in Vero E6^TMPRSS2+^ cells plateaued at 24 HPI before declining drastically after 48 HPI. Our results further demonstrated that although infectious viruses of all Omicrons kept accumulating from 6 to 24 HPI in both Vero E6 and Vero E6^TMPRSS2+^ cells, replication was much faster in Vero E6^TMPRSS2+^ cells than in Vero E6 cells (Fig. 1D). The severe cytopathic effects were observed in Vero E6^TMPRSS2+^ cells as early as 48 HPI (Fig. S1 to S5), and the violent decline of viability of Vero E6^TMPRSS2+^ cells from 24 to 48 HPI (Fig. 1E; Fig. S6) implied that cell death probably led to the gradual decrease of titers starting from 24 HPI. Moreover, the fast replication of HK.3 in Vero E6^TMPRSS2+^ cells indicated its putative strong utilization of TMPRSS2. We quantified relative TMPRSS2 and ACE2 expressions (from both *Chlorocebus sabaeus* and *Homo sapiens*) in cell lines by qRT-PCR. Vero E6^TMPRSS2+^, Caco2, and Huh-7 cells exhibited higher TMPRSS2 expressions than Vero E6 cells, while they revealed comparable expression levels of ACE2 (Fig. 1F). By comparing replication between Vero E6^TMPRSS2+^ cells and Vero E6 cells at the indicated time points, we found that the propagation of HK.3 was significantly more efficient than XBB.1.9.1 and EG.5.1 at both 12 and 24 HPI when TMPRSS2 was overexpressed (Fig. 1G). Moreover, few replication discrepancies between the two HK.3 isolates (CC1 and CC2) were demonstrated in the five cell lines (Fig. 1H). Thus, only HK.3-CC1 was used for further studies.

### Characteristics of XBB.1.9 spike mutations

**Fig 2:**
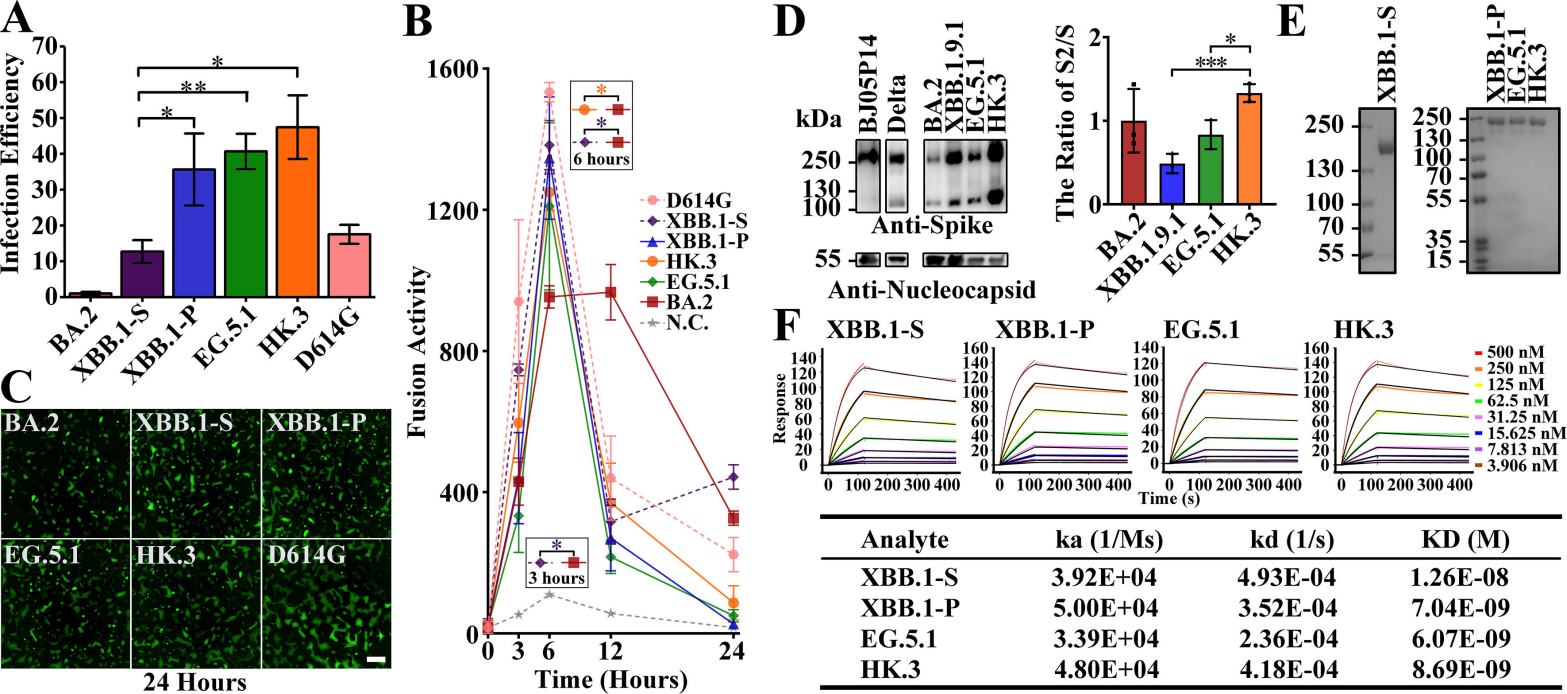
Characteristics of the spikes of XBB.1.9 subvariants. (**A**) Spike-mediated infection determined by pseudovirus assays. XBB.1-S of the XBB.1 lineage and XBB.1-P of the XBB.1.9 lineage were included. The infection efficiency of BA.2 has been set to 1 to show relative infectivity. (**B**) Spike-mediated cell‒cell fusion based on luciferase activity. BA.2 (dark red), XBB.1-P (blue), EG.5.1 (green), and HK.3 (orange) are indicated by solid lines. D614G (pink), XBB.1-S (purple), and a negative control (N.C. in gray) are indicated by dotted lines. The significance of the differences between XBB.1 variants and BA.2 is indicated in colors corresponding to the individual XBB variants, which are placed within black rectangles by the asterisks, respectively. (**C**) Spike-mediated syncytia formation (scale bar: 400 µm). (**D**) The proteolytic processing of spike protein was analyzed in authentic SARS-CoV-2 virions propagated in Vero E6^TMPRSS2+^ cells, including the ancestral strain (BJ05P14), Delta, and Omicron subvariants (BA.2, XBB.1.9.1, EG.5.1, and HK.3). Relative spike protein expression levels of BA.2, XBB.1.9.1, EG.5.1, and HK.3 virions were determined (a representative result) with an exposure time of 1 ms (left). The ratio of S2 subunit bands to full-length S protein (S2/S) was quantified (three biological replicates) using ImageJ/Fiji software (right). The ratio of BA.2 has been set to 1. (**E**) Purification of XBB.1-S, XBB.1-P, EG.5.1, and HK.3 spikes. (**F**) Comparison of the binding affinities of the XBB.1 spikes to hACE2. SPR characterization of the spike includes XBB.1-S, XBB.1-P, EG.5.1, and HK.3 interacting with hACE2. The dissociation constant is revealed above the lines. Sensorgrams depict different concentrations of ligands. Statistical analyses were conducted using Student’s *t*-test. Significances: *P* < 0.05 (*), *P* < 0.01 (**), or *P* < 0.001 (***).

The characteristics of spike proteins are regarded as key factors in *in vitro* replication, pathogenicity, and transmission (8, 14, 15). To investigate the mutation-induced characteristics of the XBB.1.9 spike protein, we first determined spike-mediated infectivity by pseudovirus-based infection. F486P in XBB.1.9 (XBB.1-P) resulted in significantly greater infectivity than that of XBB.1 with F486S (XBB.1-S) in HeLa^hACE2+^ cells at 24 h (Fig. 2A). Although the subsequent variants, EG.5.1 and HK.3, retained high infectivity, there were no significant differences between these strains and XBB.1-P, consistent with the findings of a recent study (7). The XBB.1-P, EG.5.1, and HK.3 spikes also resulted in greater infectivity than those of D614G and BA.2. The formation of syncytia is regarded as a hallmark of SARS-CoV-2-induced pathogenesis in the lungs (16, 17) and is caused by spike–ACE2 interactions on the cell surface, referred to as spike-mediated cell‒cell fusion. At 6 h post-cell contact, despite the large intragroup differences in both XBB.1-P and EG.5.1, the XBB.1-S and HK.3 spikes demonstrated significantly greater fusogenicity than that of BA.2 (Fig. 2B). The differences in the fusogenicity of the XBB.1-P, EG.5.1, and HK.3 spikes were not significant. In homology, we observed that HK.3 induced more extensive syncytia than BA.2 at 24 h, despite the semi-quantitative nature of this assessment (Fig. 2C).

The activation of spike protein processing at the S1/S2 polybasic cleavage site on the virion is positively correlated with increased infection and fusogenicity (9, 11). To directly investigate the effect of the L455F/F456L double mutation on the processing of the XBB.1 spike protein, we collected authentic SARS-CoV-2 virions grown in Vero E6^TMPRSS2+^ cells and performed an immunoblot assay; the results revealed ~250 kDa bands corresponding to the full-length spike protein and ~120 kDa bands corresponding to the S2 subunit (Fig. 2D). HK.3 with L455F/F456L double mutations showed stronger cleavage than XBB.1.9.1 and EG.5.1, and this effect was probably associated with efficient replication in the cell lines. The data demonstrate that HK.3 exhibits higher spike cleavage efficiency than XBB.1.9.1 and EG.5.1 (*P* < 0.05). Our results, consistent with those of a previous study (18), indicated that the Delta spike protein was strongly cleaved. Virions of another SARS-CoV-2 variant isolated in our laboratory (data not published, named BJ05P14), which contains a deficient furin cleavage site in the spike protein, barely exhibited spike cleavage.

Spikes of epidemic Omicron lineages have demonstrated higher binding affinities than those of previous variants (19). We purified the ectodomains of XBB.1 spikes (Fig. 2E) and measured their binding affinities to hACE2 via surface plasmon resonance (SPR). XBB.1 spike proteins with F486P, including EG.5.1 and HK.3, demonstrated lower dissociation rates than XBB.1-S and 1.5- to 2-fold higher binding affinity to hACE2 than XBB.1-S (Fig. 2F). Despite the slightly higher binding affinity to hACE2 of EG.5.1, the three proteins with F486P exhibited similar binding affinities for hACE2.

### Virological characteristics of XBB.1.9 subvariants *in vivo*

**Fig 3:**
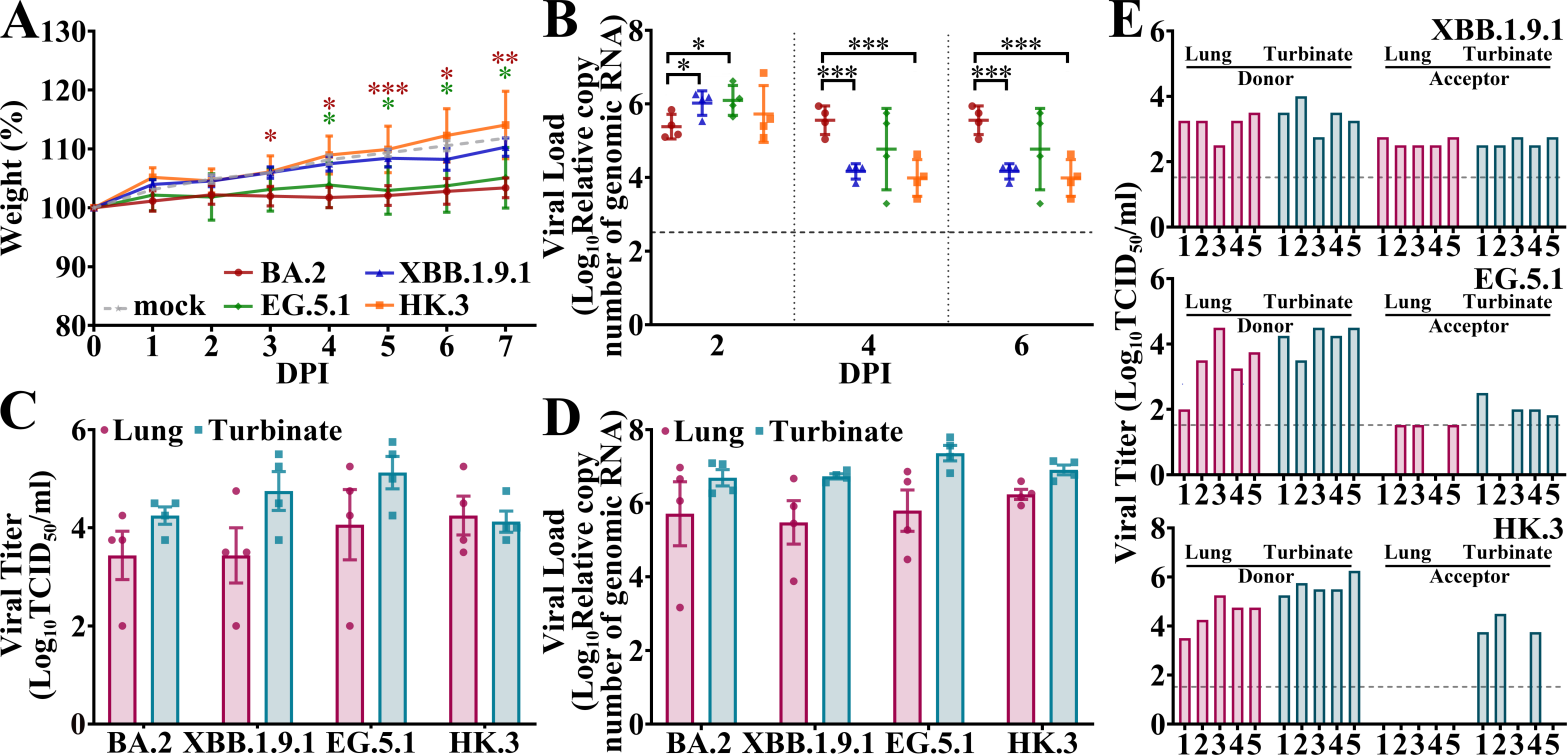
*In vivo* virological characteristics of the XBB.1.9 subvariants in wild-type hamsters. Hamsters were intranasally inoculated with BA.2, XBB.1.9.1, EG.5.1, or HK.3, and the corresponding results (**A and B**) are shown in red, blue, green, and orange, respectively (as shown in panel **A**). (**A and B**) Four hamsters per group were used to measure body weights (**A**) and relative viral loads in nasal lavages (**B**). Viral titers (**C**) and viral loads (**D**) in the lungs (dark red) or turbinates (gray) of the infected hamsters at 3 DPI. (**E**) Airborne transmission of XBB.1.9.1 (upper), EG.5.1 (middle), and HK.3 (lower) in hamsters. The viral titers of the lungs and turbinates of the inoculated donors and acceptors at 5 DPI are indicated in dark red and gray, respectively. Statistical analyses were conducted using Student’s *t*-test. Significances: *P* < 0.05 (*), *P* < 0.01 (**), or *P* < 0.001 (***).

We evaluated the pathogenicity of XBB.1.9.1, EG.5.1, and HK.3 in common wild-type Syrian hamster models. All the infected hamsters survived after the challenge. Although the BA.2- and EG.5.1-infected hamsters exhibited significantly lower weights than the mock-infected hamsters, most of the animals infected with the four Omicron isolates gained weight over the 7-day experiment (Fig. 3A). Although the XBB.1.9.1 and EG.5.1 groups exhibited greater viral loads in the nasal lavages than did the BA.2 group at 2 days post-infection (DPI), viral loads of all the XBB groups decreased very quickly compared to those in the BA.2 group (Fig. 3B). Thus, both XBB.1.9.1 and EG.5.1 infection led to lower viral loads in nasal lavages than that of BA.2. No significant differences in viral loads or viral titers were detected in the lungs or turbinates of the four groups at 3 DPI (Fig. 3C and D). According to the nasal lavage and turbinate results, all four Omicron infections resulted in large intragroup differences in viral titers in the lower airways, which is highly consistent with previous results from other groups and our group (20, 21).

Given the increasing prevalence of XBB.1.9 and its subvariants, we evaluated the airborne transmission of XBB.1.9.1, EG.5.1, and HK.3 in hamsters (Fig. 3E). High transmissibility of XBB.1.9.1 was observed in hamsters (5/5), with infectious virus detected in both nasal turbinates and lungs at 5 DPI. Interestingly, although EG.5.1 also exhibited high transmissibility in hamsters (5/5), infectious viruses were inconsistently detected in the upper and lower airways of the exposed acceptors. Specifically, lung infection of 4/5 exposed hamsters (#1, #3, #4, and #5) and turbinate infection of 3/5 hamsters (#2, #3, and #5) were demonstrated. Near-detection-limit viral titers were merely demonstrated from two acceptors (with lung of #2 and turbinate of #5). For HK.3, infectious viruses with higher titers were detected exclusively in the nasal turbinates in three of five exposed acceptors. Compared to our previous results indicating poor transmissibility of BA.2 in hamsters (1/5, exclusively detected in turbinate) (21), the transmissibility of XBB.1.9 subvariants was substantially enhanced, although the transmissibility of XBB.1.9.1 in hamsters was slightly greater than that of the other two variants.

**Fig 4:**
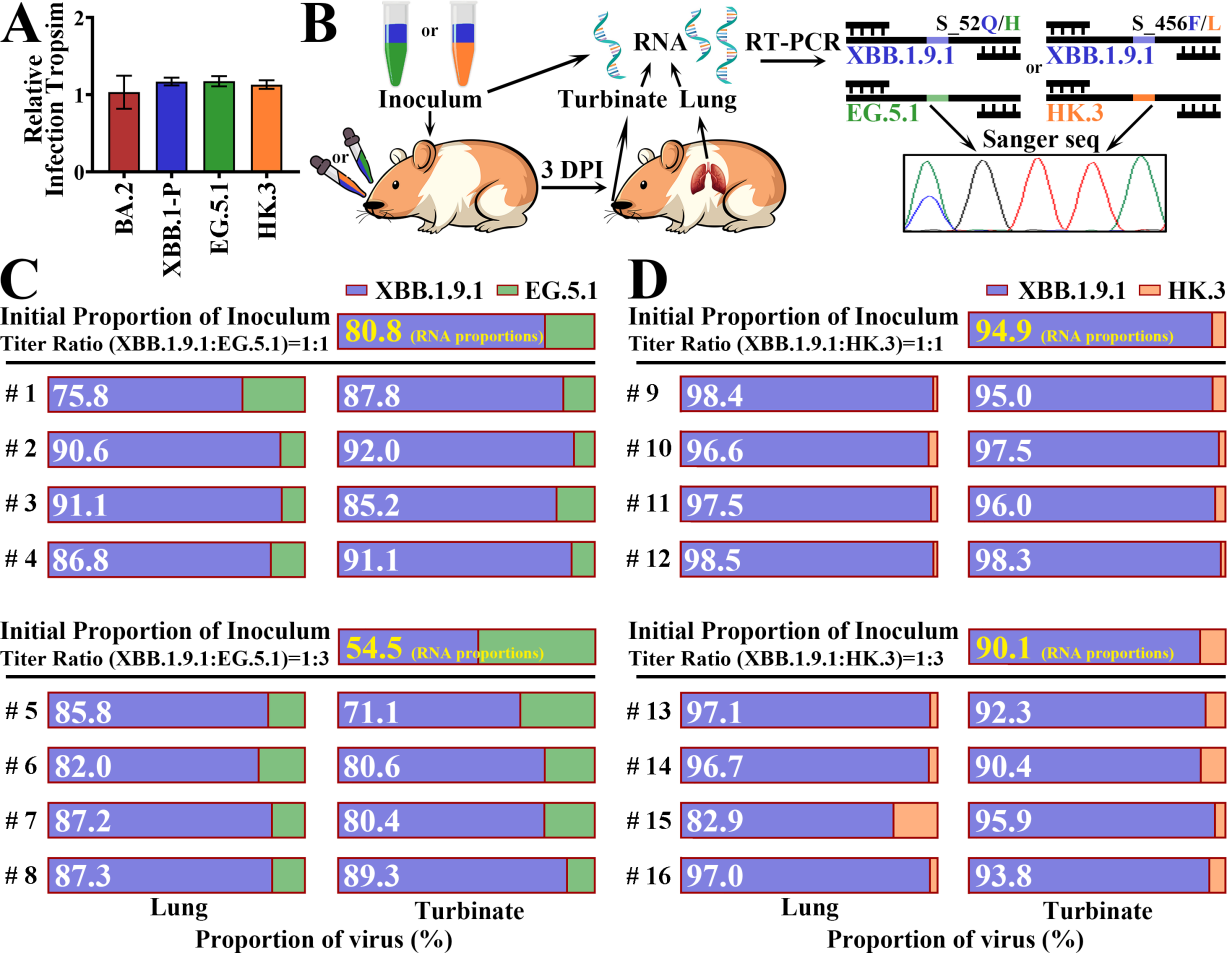
Competitive fitness of XBB.1.9.1 and EG.5.1/HK.3 in wild-type hamsters. (**A**) Relative infection tropism of spikes. The infectivity ratio of ghACE2 to hACE2 is determined as tropism. (**B**) Flow chart of competitive fitness. (**C and D**) A mixture of XBB.1.9.1 and EG.5.1 (**C**) or HK.3 (**D**) at viral titer ratios of 1:1 (upper panel) or 1:3 (lower panel) was inoculated into hamsters. The RNA proportion of XBB.1.9.1 in the mixture was shown by numbers in the bars. Firstly, the RNA proportion of XBB.1.9.1 in initial inoculum was 80.8% or 54.5% (**C**) and 94.9% or 90.1% (**D**) which was displayed on the right of the initial proportion (yellow number). Secondly, the RNA proportion of XBB.1.9.1 in tissue samples (3 DPI) was shown in the bars (white number) below the horizontal of each figure grouping. The area in the bar means the RNA proportions of XBB.1.9.1 (blue) and EG.5.1 (green) or HK.3 (orange). Tissue samples are the lung and turbinate: lung (left) and turbinate (right).

Given the relatively efficient transmission of EG.5 and HK.3 in humans (7) and their slightly lower transmissibility in hamsters than that of XBB.1.9.1, we compared the *in vivo* fitness of XBB.1.9.1 and EG.5.1/HK.3 in hamsters. However, because of the differences in ACE2 receptors, the comparative transmissibility or fitness of variants revealed in hamsters may not reflect their properties in humans. We first evaluated spike infectivity tropism of golden hamster ACE2 (ghACE2) compared to that of hACE2 by measuring the infectivity of pseudovirus in HEK-293T cells (Fig. 4A). The result of the spikes of multiple XBB.1.9 and even BA.2 demonstrated similar tropism. Therefore, the differences between ghACE2 and hACE2 utilization of variants do not induce a bias in evaluating XBB transmission, at least in hamster models. The indicated two variants (XBB.1.9.1 and EG.5.1/HK.3) were mixed and intranasally inoculated into hamsters. RNA proportions of the compared variants in the initial inoculum and in hamster tissues were measured using RT-PCR followed by Sanger sequencing (Fig. 4B) to assess the relative fitness. For animals inoculated with an XBB.1.9.1 and EG.5.1 mixture in a 1:1 ratio, the RNA proportion of XBB.1.9.1 largely increased from 80.8% (in inoculum) to more than 85% in the lungs and turbinates of each hamster (Fig. 4C, upper panel), except in the lung of hamster 1 (75.8%), indicating the fitness of XBB.1.9.1 RNA. When an XBB.1.9.1 and EG.5.1 mixture of a 1:3 viral titer ratio was inoculated, an obvious fitness of XBB.1.9.1 was observed in both lungs and turbinates of hamsters (Fig. 4C, lower panel). Similarly, XBB.1.9.1 also outcompeted HK.3 in the lungs and turbinates of hamsters, except in the lung of hamster 15 (Fig. 4D). Taken together, these results suggest that XBB.1.9.1 may have greater replicative fitness than EG.5.1 and HK.3 in hamsters, especially in the upper respiratory tract.

**Fig 5:**
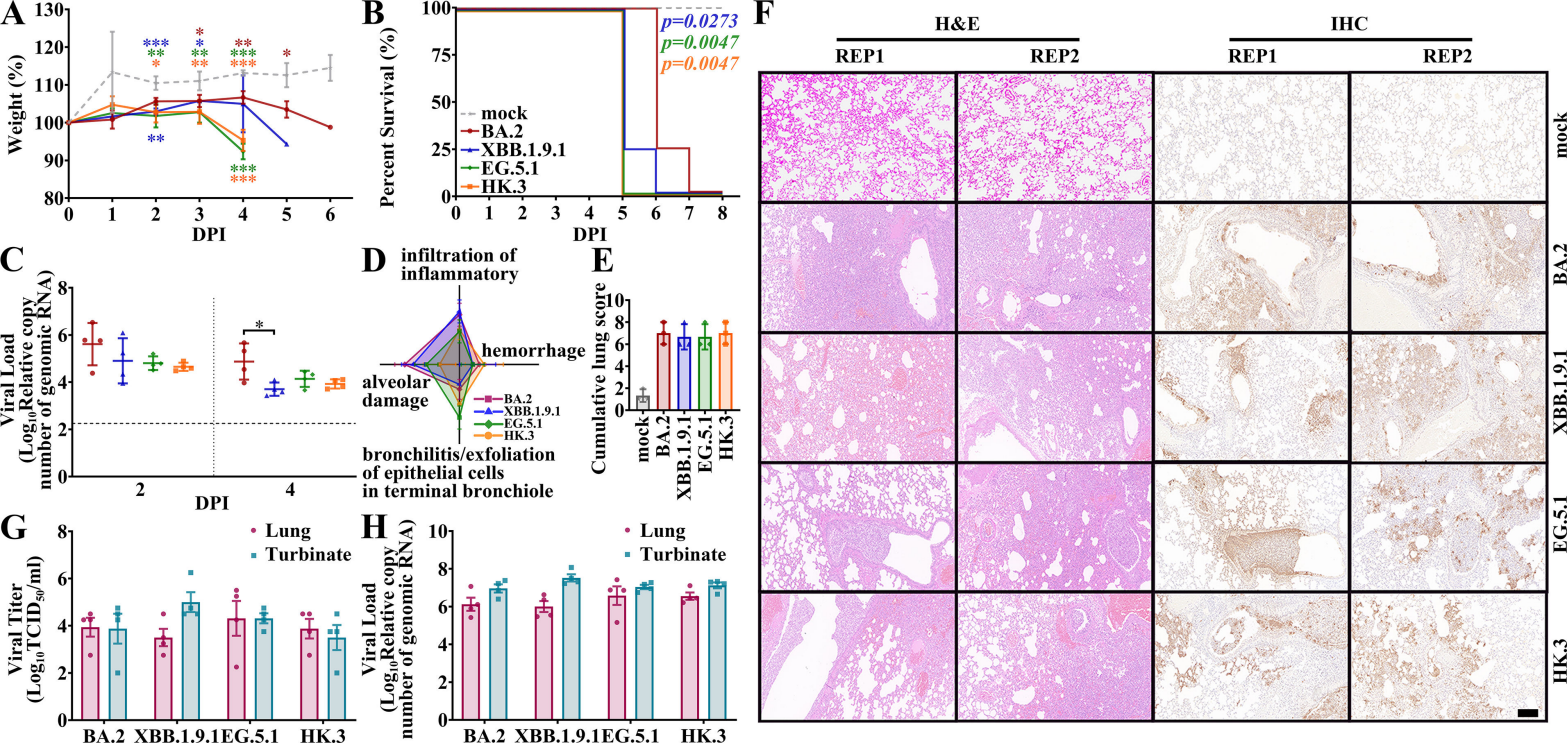
*In vivo* virological characteristics of XBB.1.9 subvariants in K18-hACE2 hamsters. K18-hACE2 hamsters were intranasally inoculated with BA.2, XBB.1.9.1, EG.5.1, or HK.3. Four hamsters per group were used to measure the various parameters (**A, B, and C**). Four hamsters per group were euthanized at 3 DPI and used for data collection (**D–H**). The data (in A to E) of the mock, BA.2, XBB.1.9.1, EG.5.1, and HK.3 groups are shown in gray, red, blue, green, and orange, respectively (as shown in panel **A**). (**A**) Body weights of the infected hamsters. Significant differences between the mock group and each infected group are revealed above the lines using asterisks in the colors corresponding to the respective infected group. (**B**) Percentage survival of the infected hamsters. Survival differences between multiple XBB.1.9 variants and BA.2 were analyzed using a Log-rank (Mantel-Cox) test with significance displayed in colors corresponding to the individual XBB.1.9 variant. (**C**) Viral loads in the nasal lavages of hamsters. The viral load baseline is indicated by dotted gray lines. (**D and E**) Radar chart of pathology (**D**) and pathology scores (**E**) of the infected lungs of hACE2 hamsters. The average of BA.2 (dark red), XBB.1.9.1 (blue), EG.5.1 (green), and HK.3 (orange) infected hamster (of 3–4 individuals) was indicated. (**F**) H&E staining and IHC images of the lungs of the infected hamsters. The lungs of two infected individuals in each group, namely, repetition 1 (REP1) and repetition 2 (REP2), are shown. The time point of tissue samples corresponds to 3 DPI. The scale bar represents 100 µm. (**G and H**) Viral titers (**G**) and viral loads (**H**) in the lungs (dark red) or turbinates (gray) of the infected hamsters. Statistical analyses were conducted using Student’s *t*-test. Significances: *P* < 0.05 (*), *P* < 0.01 (**), or *P* < 0.001 (***).

We then investigated the replication and pathogenicity of the XBB.1.9 subvariants using previously reported Omicron-lethal H11-K18-hACE2 transgenic hamster models (21, 22). Similar to the results of BA.2 and BA.5.2.48 infection (21), all three XBB.1.9-infected hACE2 hamster groups exhibited high mortality (Fig. 5A and B). Compared to mock infection, XBB.1.9, EG.5.1, and HK.3 infection induced significant body weight changes as early as 2 DPI, earlier than that observed for BA.2 (at 3 DPI). Weight loss was significantly induced exclusively by EG.5.1 and HK.3 infection, which was revealed just before lethality at 4 DPI. Specifically, infection with both EG.5.1 and HK.3 induced 100% lethality as early as 5 DPI, while infection with XBB.1.9.1 induced 75% lethality. BA.2 infection led to 75% and 100% lethality at 6 and 7 DPI, respectively. Both the mortality and body weight changes indicated greater pathogenicity of XBB.1.9.1, EG.5.1, and HK.3 than that of the parental BA.2. The XBB.1.9.1 group exhibited lower viral loads in nasal lavages than the BA.2 group at 4 DPI, but no significant differences were found among the XBB.1.9.1, EG.5.1, and HK.3 groups (Fig. 5C). To evaluate the pathological features and determine the infected areas in the lungs, we performed H&E staining and anti-nucleocapsid immunohistochemistry (IHC) of the hamster lungs. We observed universal alveolar wall thickening and infiltration of inflammatory cells in the alveolar spaces and bronchial mucosal epithelial cells in all groups. The viruses caused similar infections in multiple areas of the lungs, from the epithelium of the terminal bronchiole to the alveoli. Moreover, despite comparable pathology scores among the different groups at 3 DPI (Fig. 5D through F), infection with BA.2 and XBB.1.9.1 led to greater inflammatory infiltration (BA.2: 2.50 and XBB.1.9.1: 2.33; compared to HK.3: 1.75 and EG.5.1: 1.67), while EG.5.1 and HK.3 infection tended to cause a large amount of nucleocapsid-positive exfoliation of epithelial cells in terminal bronchioles, forming widespread airway obstructions (HK.3: 2.00 and EG.5.1: 2.33 compared to BA.2: 1.25 and XBB.1.9.1: 1.00). Similar to the results in wild-type hamsters, no significant differences in viral loads or viral titers were detected in the lungs or turbinates of the four groups of transgenic hamsters at 3 DPI (Fig. 5G and H).

## DISCUSSION

In comparison to previous VOCs, Omicron BA.1 and BA.2 are less likely to induce pneumonia and other severe symptoms in COVID-19 patients (23, 24), which is consistent with their attenuated pathogenicity revealed in animal models; similarly, BA.5 also failed to significantly increase pathogenicity compared to BA.2 (20, 25, 26). However, the attenuation of pathogenicity by Omicron is not continuous. We previously reported that a subvariant of BA.5, BA.5.2.48, demonstrated greater pathogenicity than BA.2 in H11-K18-hACE2 rodents (21). In this study, H11-K18-hACE2 hamsters infected with multiple XBB.1.9 subvariants (especially EG.5.1 and HK.3) revealed earlier mortality and significantly greater weight loss than did the hamsters infected with BA.2. We observed more exfoliation of epithelial cells in the bronchioles of EG.5.1- and HK.3-infected lungs than in those of BA.2, which formed severe obstacles in airway and probably impeded normal respiration (Fig. 5D). This is regarded as one of the key factors contributing to lethality. The spike F486P mutation in both XBB.1.9.1 and XBB.1.9.2 sublineages, including EG.5.1 and HK.3, was reported to be associated with increased pathogenicity in XBB.1 (27), which is probably attributed to its higher infectivity and hACE2 binding affinity of the spike protein reported, as reported by us and others (28).

In contrast to the limited airborne transmission of BA.1 and BA.2 reported by other groups and our group (21, 29), XBB.1.9.1, EG.5.1, and HK.3 demonstrated efficient transmissibility in hamster models (Fig. 3E), which is probably a key factor contributing to the prevalence. We co-infected hamsters with viral mixtures in different ratios of titers (1:1 or 1:3, in Fig. 4C and D). However, due to factors such as RNA packaging efficiency and the presence of non-infectious particles, the proportion of viral titer does not directly equate to proportion of viral RNA. Therefore, we explicitly annotated the initial proportion of viral mixtures’ RNA that displayed on the right side of the viral titer (each figure group) and utilized proportions of RNA as the primary metric for determining the competitive fitness of variants *in vivo*. As a result, XBB.1.9.1 exhibited stronger fitness in the upper airways, which explained to some extent its slightly greater transmissibility compared with EG.5.1 and HK.3.

Interestingly, although HK.3 reveals faster replication than other Omicrons in multiple cell lines (Fig. 1C and D), partially owing to its higher TMPRSS2 utilization and enhanced spike processing (Fig. 1G and 2D), it failed to demonstrate enhanced pathogenicity and airborne transmission in animal models, which implies that results of *in vitro* and *in vivo* virological features are not entirely consistent. Moreover, as another key fact affecting prevalence of multiple variants, immune evasion should also be taken into consideration. Besides the moderate airborne transmission, the prevalence of HK.3 might be partly attributed to its enhanced immune evasion (7).

## MATERIALS AND METHODS

### Sequences identification information and epidemiological

Viral genomic sequence identification and phylogenetic analysis pipelines were sourced from two authoritative public repositories. CoV-Lineages (https://cov-lineages.org) is the official platform for SARS-CoV-2 lineage classification under the Pango nomenclature system. Outbreak.info (https://outbreak.info) is a WHO-collaborating genomic surveillance database. All data queries and analytical workflows were executed on September 26, 2023. A SARS-CoV-2 variant carrying both the C11956T and Spike_F486P mutations has been designated as the novel lineage XBB.1.9.1. Another variant with mutations at A16878T, A27507C, and Spike_F486P has been classified as XBB.1.9.2. The EG.5.1 sublineages (XBB.1.9.2.5.1) emerged from XBB.1.9.2 through the acquisition of two additional spike mutations: Q52H and F456L. HK.3 (XBB.1.9.2.5.1.1.3), defined by the Spike_F455L substitution (https://github.com/), was analyzed for its lineage distribution. The lineage relative frequencies over time per month in China (CHN), the United States of America (USA), Europe (EUP), and the Republic of Korea (ROK) through 31 March 2024 were downloaded from GISAID (https://www.epicov.org) on 10 April 2024.

### Virus

The four SARS-CoV-2 Omicron XBB.1.9 subvariants used in this study were as follows: an XBB.1.9.1 isolate (hCoV-19/Jilin/JSY-CC8/2023, GISAID Accession No. EPI_ISL_18908494), which contains the extra mutations A10323G (NSP5_K90R), C11750T (NSP6_L260F), G25352T (Spike_V1264L), and G26634T (M_A38S); an EG.5.1 isolate (hCoV-19/Jilin/JSY-CC9/2023, GISAID Accession No. EPI_ISL_18908495), which contains the extra mutations T9037A (NSP4_D161E), T18285G (NSP14_H82Q), and G23587C (Spike_Q675H); and two HK.3 isolates, referred to as HK.3-CC1 (hCoV-19/Jilin/JSY-CC11/2023, GISAID Accession No. EPI_ISL_18908496) containing the extra mutation GTT26284-26286del (E_V14del) and HK.3-CC2 (hCoV-19/Jilin/JSY-CC12/2023, GISAID Accession No. EPI_ISL_18908497) containing the extra mutations G11083T (NSP6_L37F), C24912T (Spike_T1117I), and GTT26284-26286del (E_V14del). A BA.2 isolate (hCoV-19/Jilin/JSY-CC5/2022, GISAID Accession No. EPI_ISL_18435548) was also used. Clinical specimens (nasal swabs with DMEM) were inoculated into Vero E6 cells cultured in 6-well plates with DMEM containing 2% FBS. At 72 HPI, supernatant was transferred to freshly cultured Vero E6 cells for continued propagation. The remaining supernatant was archived at −80°C. Distinct cytopathic effects were observed by the second passage (P2). The supernatant of P2 was used for RNA extraction, qRT-PCR quantification, and genome sequencing. Re-sequencing of the viral spike was conducted prior to the formal experiments (after two additional passages) to confirm the absence of cell culture–adapted mutations.

### Viral titer and cell viability

Cells seeded in 6-well or 96-well plates were infected with virus at a multiplicity of infection (MOI) of 0.01. Following a 1-hour incubation at 37°C/5% CO_2_, the virus-containing medium was aspirated, and the cells were washed twice with PBS and maintained in DMEM supplemented with 2% FBS. At 6, 12, 24, 48, 72, and 96 HPI, 100 µL or 140 µL supernatant aliquots (from 6-well plates) were collected for either viral titer or load quantification. Viral titers were measured using TCID_50_ assays as previously described (21) with a baseline of 33 TCID_50_/mL. Cell viability in 96-well plates at the intended time points was measured using a Cell Counting Kit-8 (MedChemExpress, HY-K0301).

### Plasmids, PCR, and qRT‒PCR

The sequences encoding the D614G, BA.2, XBB.1-S, XBB.1-P, EG.5.1, and HK.3 spike proteins lacking the C-terminal 19 amino acids (spike-D19) were synthesized and cloned into a pcDNA3 vector (to yield multiple pcDNA3-spike plasmids). The sequences encoding the hACE2 or ghACE2 were synthesized and cloned into a pcDNA3 vector (to yield plasmids pcDNA3-hACE2 and pcDNA3-ghACE2). Codon-optimized XBB.1-P, EG.5.1, and HK.3 spike ectodomains with a furin cleavage site mutation (682RRAR685 mutated to 682GSAS685), a “HexaPro” modification, and a 6 × His-StrepII tag were synthesized and ligated into a pcDNA3 plasmid to generate multiple pcDNA3-spike-HexaPro plasmids. The dual split protein (DSP) plasmids pDSP1-7 and pDSP8-11, which encode the split Renilla luciferase and GFP genes, were previously described (21).

Viral RNA extraction was performed using a QIAamp Viral RNA Mini Kit (Qiagen). One-step qRT‒PCR was used to determine the relative copy number of genomic RNA of SARS-CoV-2, as previously described (30). To distinguish replicating viral genomes in infected samples from those in aerosol-contaminated samples, we used the average viral load in samples collected from uninfected animals (mock groups) as a detection baseline in qRT‒PCR results of *in vivo* experiment.

Relative RNA expressions of ACE2 and TMPRSS2 were determined by qRT-PCR using iTaq Universal SYBR Green Supermix (Biorad), in which primer pairs (Table S1) were designed to target conserved regions of TMPRSS2, ACE2, or GAPDH (as a housekeeping reference gene) RNA from both *Chlorocebus sabaeus* and *Homo sapiens*. Cellular RNA extraction was performed using a RNAsimple Total RNA Kit (Tiangen) and reverse-transcribed into cDNA using SuperScript IV Reverse Transcriptase (Thermo). Data were analyzed by calculating Log_10_(2^(−ΔΔct)).

### Cells, transfection, protein purification, and SPR

Vero E6 cells, Vero E6^TMPRSS2+^ cells, HeLa^hACE2+^ cells, Huh-7 cells, Caco2 cells, HEK-293T cells, and HEK-293F cells were cultured and transfected using either Lipofectamine 3000 (Thermo) or polyethylenimine (Polysciences) as previously described (30, 31). The Vero E6^TMPRSS2+^ cells were obtained from the Japanese Collection of Research Bioresources Cell Bank, while all other cell lines were maintained in-house. HEK-293F cells were transfected with pcDNA3-spike-HexaPro for 5 days. Spike proteins in culture medium were purified with gravity flow columns using Strep-Tactin XT (IBA Life Sciences), concentrated to 0.5 mg/mL with ultrafiltration tubes (Millipore), and stored at −80 ℃ before use. The purified proteins were analyzed on 6% SDS‒PAGE gels with Coomassie blue staining. The SPR experiments were performed using a Biacore T200 (Cytiva). All assays were performed with 1 × HEPES running buffer (10 mM HEPES, 150 mM NaCl, 3 mM EDTA, and 0.005% Tween-20, pH 7.4) at 25℃. For the determination of the binding kinetics between the spike protein and hACE2 proteins, a Protein A sensor chip (Cytiva) was used. The hACE2 protein with an Fc tag (Acro Biosystems) was immobilized onto the sample flow cell of the sensor chip. The reference flow cell was left blank. Each spike protein was injected over the two flow cells at eight concentrations, prepared by serial twofold dilutions ranging from 3.906 nM to 500 nM, at a flow rate of 30 µL/min using a single-cycle kinetics program. All the data were fitted to a 1:1 binding model using Biacore T200 Evaluation Software 3.1.

### Pseudovirus production and infection

The vesicular stomatitis virus (VSV) pseudotyped virus deficient in the G gene (ΔG), bearing a firefly luciferase reporter gene and the VSV-G glycoprotein for infection (VSV-Luciferase-ΔG*G, Brain Case), was passaged in HEK-293T cells transfected with the pMD2.G plasmid. For the production of spike-glycoprotein-bearing pseudovirus (VSV-Luciferase-ΔG*Spike), HEK-293T cells were infected with VSV-Luciferase-ΔG*G at an MOI of 0.1 after transfection with pcDNA3-spike plasmids (or pcDNA3 as a negative control) and washed twice with DMEM after 2 h. Media containing pseudoviruses at 36 HPI were centrifuged at 500 × *g* for 10 min, and the supernatant was stored at −80°C. For the measurement of spike-mediated infection, 2 × 10^4^ HeLa^hACE2+^ cells or HEK-293T cells transiently transfected with pcDNA3-hACE2 or pcDNA3-ghACE2 plasmids were incubated with 100 µL pseudovirus in 96-well culture plates, washed once with DMEM after 24 h, and then mixed with 100 µL of luciferase substrate (PerkinElmer). Luminescence was detected using an Infinite 200 Pro plate reader (Tecan) as spike-mediated infectivity.

### Cell‒cell fusion

Spike-mediated cell‒cell fusion was determined based on the split proteins DSP8-11 and DSP1-7, which were expressed in effector and target cells, respectively. For the preparation of effector cells, HEK293T cells in 12-well plates were cotransfected with 1 µg of pcDNA3-spike (or pcDNA3 as a negative control) and 1 µg of pDSP8-11. For the preparation of target cells, HeLa^hACE2+^ cells in 6-well plates were transfected with pDSP1-7 (2.5 µg). A total of 1.3 × 10^5^ effector cells were transferred to 96-well plates at 24 h post-transfection. After an additional 24 h, the target cells were incubated with EnduRen live cell substrate (Promega) diluted 1:250. After detachment, 100 µL of 2.6 × 10^5^ targeted cells were added to wells containing effector cells. Luminescence was measured at the indicated time points using an Infinite 200 Pro plate reader (Tecan), and fusion activity was assessed. Cell images were taken using an APX100 microscope. Imaging parameters were as follows: 488 nm excitation, BP 510–550 nm emission, and an exposure time of 100 ms.

### Animal experiments

Eight-week-old male wild-type Syrian hamsters (Charles River) or 12-week-old male H11-K18-hACE2 Syrian hamsters (State Key Laboratory of Reproductive Medicine and Offspring Health, China) were intranasally inoculated with the indicated viruses at a dose of 1,000 TCID_50_ (in 100 µL). Each infected group included four animals in the body weight subgroup and four animals in the euthanized subgroup for tissue sample collection.

For coinfection studies, XBB.1.9.1 was mixed with either EG.5.1 or HK.3 in the indicated viral titer ratio, and the virus mixture (a total of 1,000 TCID_50_ in 100 µL) was inoculated into six-week-old male wild-type Syrian hamsters (Charles River). Tissue samples were collected at 3 DPI. Following total RNA extraction, cDNA was synthesized by reverse transcription using the Maxima H Minus First Strand cDNA Synthesis Kit (QIAGEN). Spike gene fragments of the XBB.1.9.1 and EG.5.1/HK.3 strains were then PCR-amplified from the cDNA using KAPA HiFi HotStart ReadyMix (Roche). The PCR amplicons underwent Sanger sequencing, and the relative RNA proportions of distinct viral strains were determined based on the geometric mean of peak height ratios in the sequencing chromatograms. Identical amplification and sequencing primers were employed to detect viral RNA within tissue samples. The binding sites for these primers are conserved across the viral templates, exhibiting identical binding regions and nucleotide sequences. Consequently, consistent amplification and sequencing efficiency were observed. The primers used are listed in Table S1.

For the airborne transmission study between hamsters, five six-week-old male hamsters were intranasally inoculated with each of the indicated viruses at a dose of 1,000 TCID_50_ (in 100 µL). After 24 h, each infected donor hamster was cohoused with one corresponding acceptor hamster in isolation devices. The propagation cages containing the donor and acceptor were separated by 3 cm to prevent direct contact. The inoculated hamsters were placed in front of the isolator unit, which provides unidirectional airflow. Tissue samples were collected 3 days after infection for the donor hamsters or 3 days after the initial cohousing for the exposed acceptors.

For all animals, intranasal inoculation and euthanasia were performed under isoflurane anesthesia.

### Histopathology

The lungs of the animals were fixed in 4% paraformaldehyde in phosphate-buffered saline (PBS) and processed for paraffin embedding. The paraffin blocks were sliced into 3 µm thick sections and mounted on silane-coated glass slides, which were subsequently subjected to H&E staining for histopathological examination. Panoramic images of the digital slides were taken using a Pannoramic MIDI (3DHISTECH). Pathological features, including (i) bronchiolitis or exfoliation of epithelial cells in terminal bronchioles; (ii) hemorrhage; (iii) alveolar damage, including alveolar thickening or disappearance; and (iv) inflammatory infiltration, were scored using a four-tiered system as 0 (negative), 1 (weak), 2 (moderate), or 3 (severe).

### Immunoblotting, immunohistochemistry, and immunofluorescence

Virions of SARS-CoV-2 in clear supernatant (10 mL) were inactivated by adding 0.1% paraformaldehyde (final concentration) for 12 h at 4°C and then concentrated by ultracentrifugation at 90,000 × *g* for 2 h using a P80A rotor (Hettich). The pellets were resuspended in 30 µL of RIPA buffer. The samples were separated via 4–12% SDS‒PAGE gels and transferred to PVDF membranes. After the membranes were blocked with 5% milk, they were blotted with primary antibodies, incubated with horseradish peroxidase-conjugated secondary antibodies, and visualized with a chemiluminescent reagent as previously described (21). Primary antibodies were diluted in 5% BSA (bovine serum albumin) and 0.5% NaN3 in PBS, while secondary antibodies were diluted in 1% BSA in PBST. Quantification of band intensity was determined using ImageJ (NIH, Bethesda, MD). For IHC, tissue sections were processed for IHC using a primary antibody followed by a horseradish peroxidase-conjugated secondary antibody, and finally stained with a DAB substrate kit (Solarbio). Panoramic images of the digital slides were taken using an SQS-40R slide scan system (Shengqiang Technology). Immunofluorescence was performed as previously described (21). The antibodies used are listed in Table S2.

### Statistical analysis

All data are presented as the means ± SDs. Comparisons were performed using Student’s *t*-tests. Significance was defined as *P* < 0.05 (*), *P* < 0.01 (**), or *P* < 0.001 (***) and was indicated either in the figures or mentioned separately in the text. The number of repeats is specified in individual panels using discrete points. All presented data represent biological replicates.

## Data Availability

The original data that support the findings of this study are available from the corresponding author upon reasonable request.

## SUPPLEMENTAL MATERIAL

Supplemental material jvi.01342-25-s0001.docx Figures S1 to S8; Tables S1 and S2.

ASM does not own the copyrights to Supplemental Material that may be linked to, or accessed through, an article. The authors have granted ASM a non-exclusive, world-wide license to publish the Supplemental Material files. Please contact the corresponding author directly for reuse.
