# Supplementary material for: Increased pathogenicity and transmission of SARS-CoV-2 Omicron XBB.1.9 subvariants, including HK.3 and EG.5.1, relative to BA.2
Source: J Virol. 2025 Nov 18;99(12):e01342-25. doi: 10.1128/jvi.01342-25 (PMC12724380; doi:10.1128/jvi.01342-25)
Supplement: Supplemental material — Figures S1 to S8; Tables S1 and S2. [file jvi.01342-25-s0001.docx]

**Supplemental Material of**

**Increased pathogenicity and transmission of SARS-CoV-2 Omicron XBB.1.9 subvariants, including HK.3 and EG.5.1, relative to BA.2**

Qiushi Jin^1, 2, a^, Ruixue Liu^1, 3, a^, Wenqi Wang^1, 4, a^, Jichen Xie^5, a^, Fang Yan^1, 7^, Tiecheng Wang^1^, Haiyang Xiang^1^, Xianzhu Xia^1, 2, 6^, Jianmin Li^5, *^, Xuefeng Wang^1, *^, Yuwei Gao^1, 3, *^

1. Changchun Veterinary Research Institute, Chinese Academy of Agricultural Sciences, Changchun, China

2. College of Veterinary Medicine, Northeast Agricultural University, Harbin, China

3. College of Veterinary Medicine, Shanxi Agricultural University, Jinzhong, China

4. College of life sciences, Northeast Normal University, Changchun, China

5. State Key Laboratory of Reproductive Medicine and Offspring Health, Jiangsu Laboratory Animal Center, Jiangsu Animal Experimental Center of Medicine and Pharmacy, Department of Cell Biology, Animal Core facility, Key Laboratory of Model Animal, Collaborative Innovation Center for Cardiovascular Disease Translational Medicine, National Vaccine Innovation Platform, Nanjing Medical University, Nanjing, China

6. Jiangsu Co-innovation Center for Prevention and Control of Important Animal Infectious Diseases and Zoonoses, Yangzhou University, Yangzhou, China

7. College of Wildlife and Nature Reserves, Northeast Forestry University, Harbin, China

^a^These authors contributed equally to the work.

Correspondence: yuwei0901@outlook.com (YG), xuefeng_wangNUDT@outlook.com (XW) and Jianminli@njmu.edu.cn (JL)

Keywords: SARS-CoV-2, Omicron, HK.3, XBB.1.9, transmission


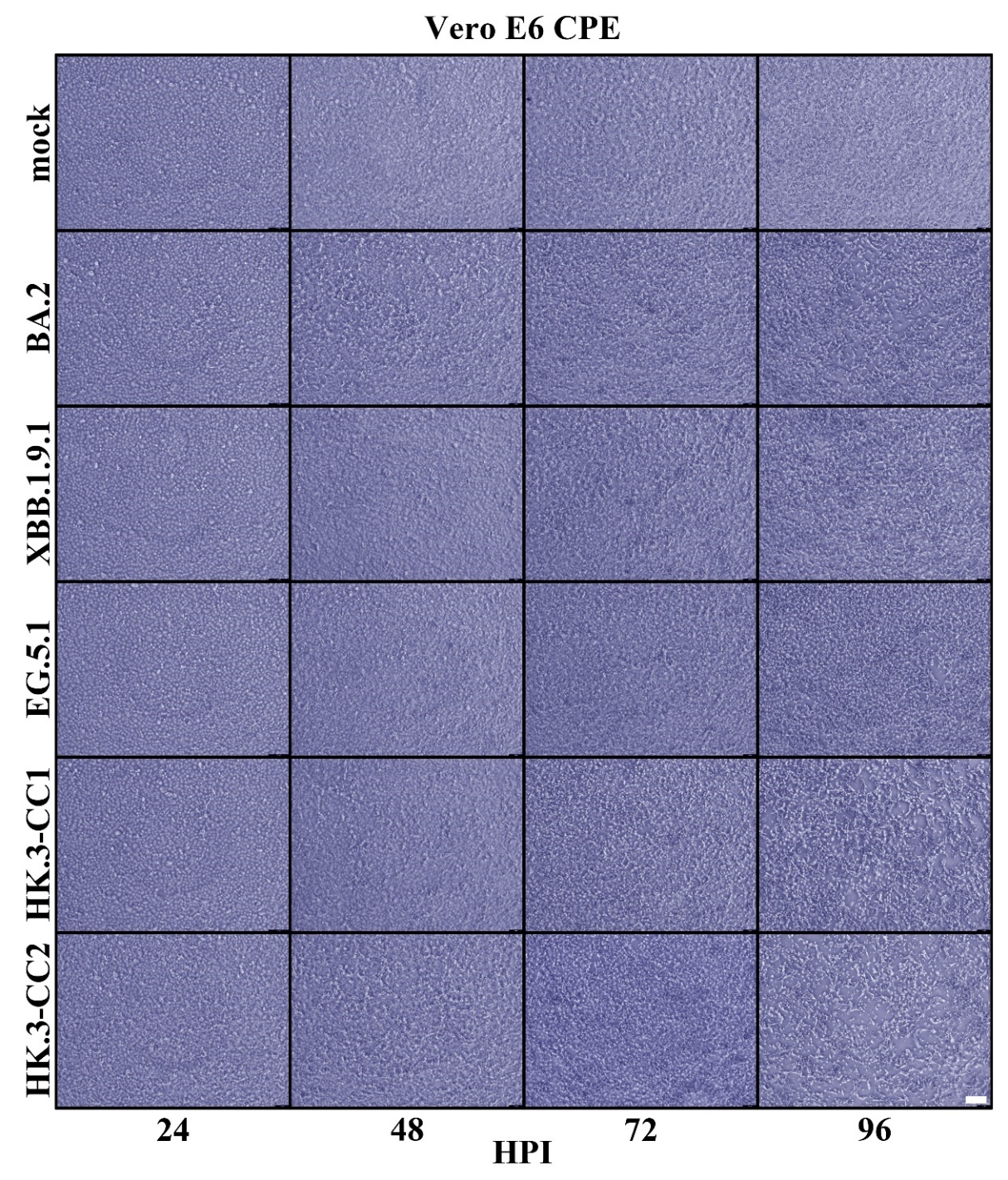


Figure S1. The image of distinct cytopathic effects for Vero E6 cells. Representative images depicting the cytopathic effects in Vero E6 following BA.2 and XBB.1.9 subvariants infection are shown from 24 to 96 HPI. Images were captured at 40× magnification; scale bar = 75 µm.


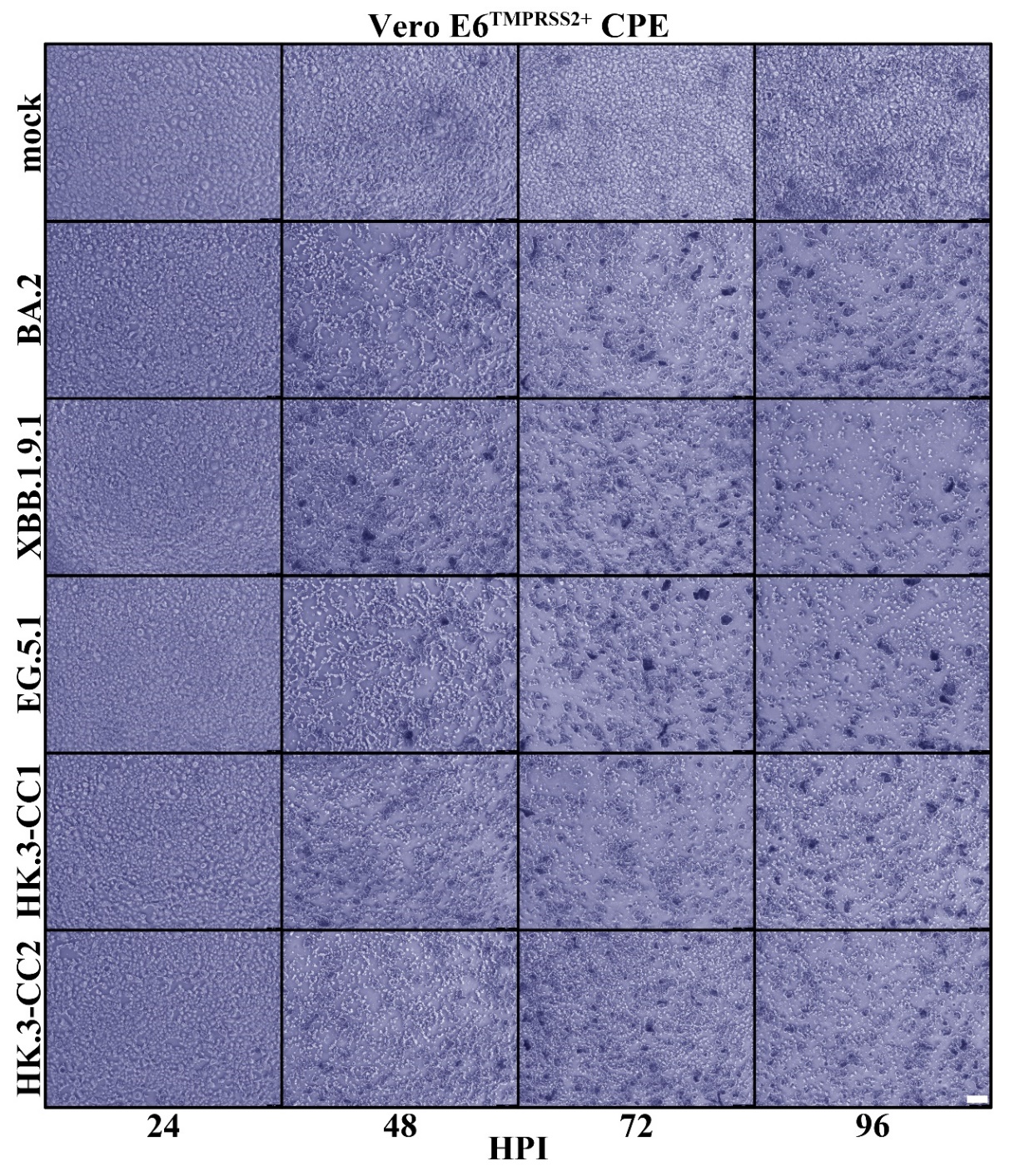


Figure S2. The image of distinct cytopathic effects for Vero E6^TMPRSS2+^ cells. Representative images depicting the cytopathic effects in Vero E6^TMPRSS2+^ following BA.2 and XBB.1.9 subvariants infection are shown from 24 to 96 HPI. Images were captured at 40× magnification; scale bar = 75 µm.


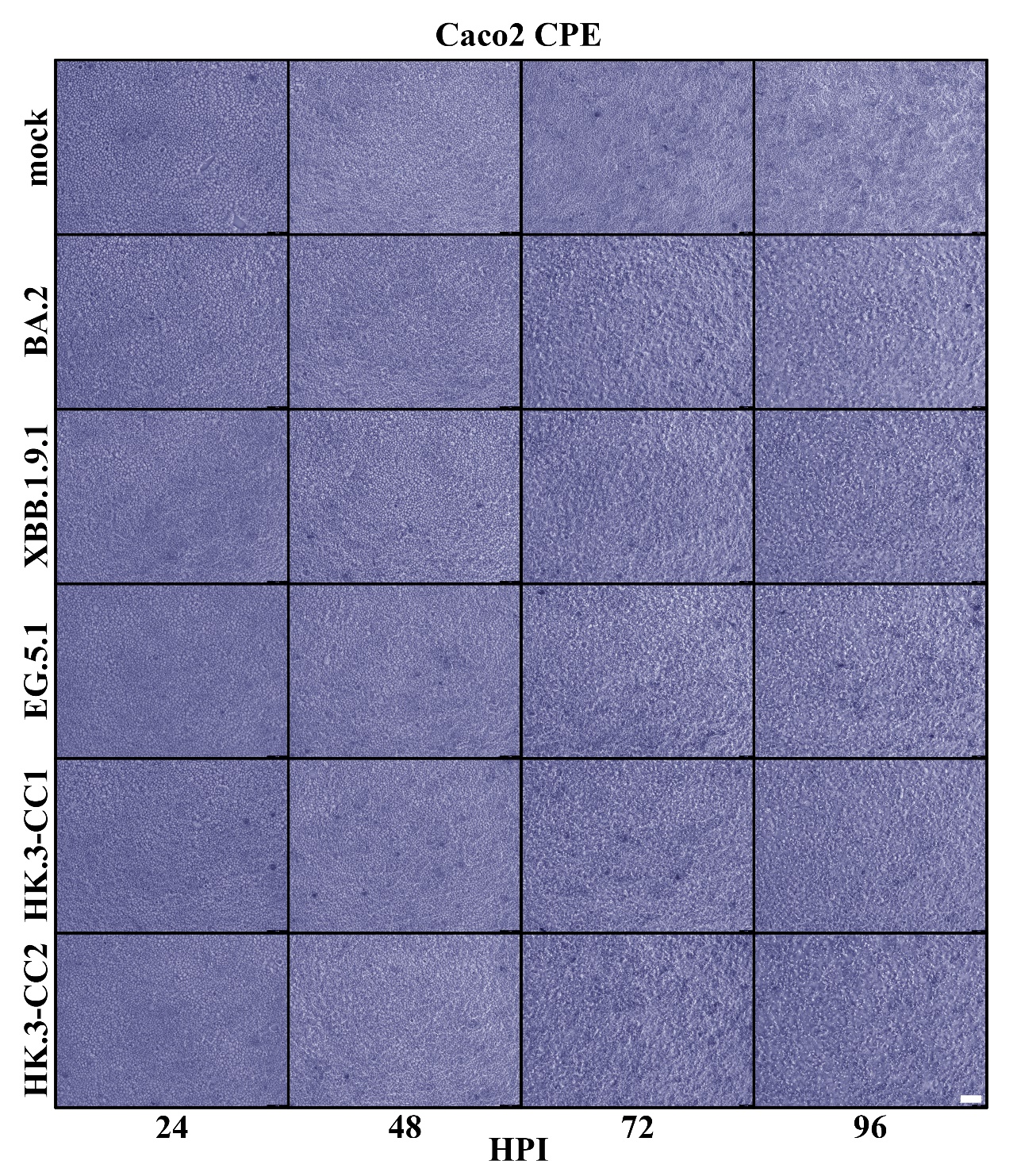


Figure S3. The image of distinct cytopathic effects for Caco2 cells. Representative images depicting the cytopathic effects in Caco2 following BA.2 and XBB.1.9 subvariants infection are shown from 24 to 96 HPI. Images were captured at 40× magnification; scale bar = 75 µm.


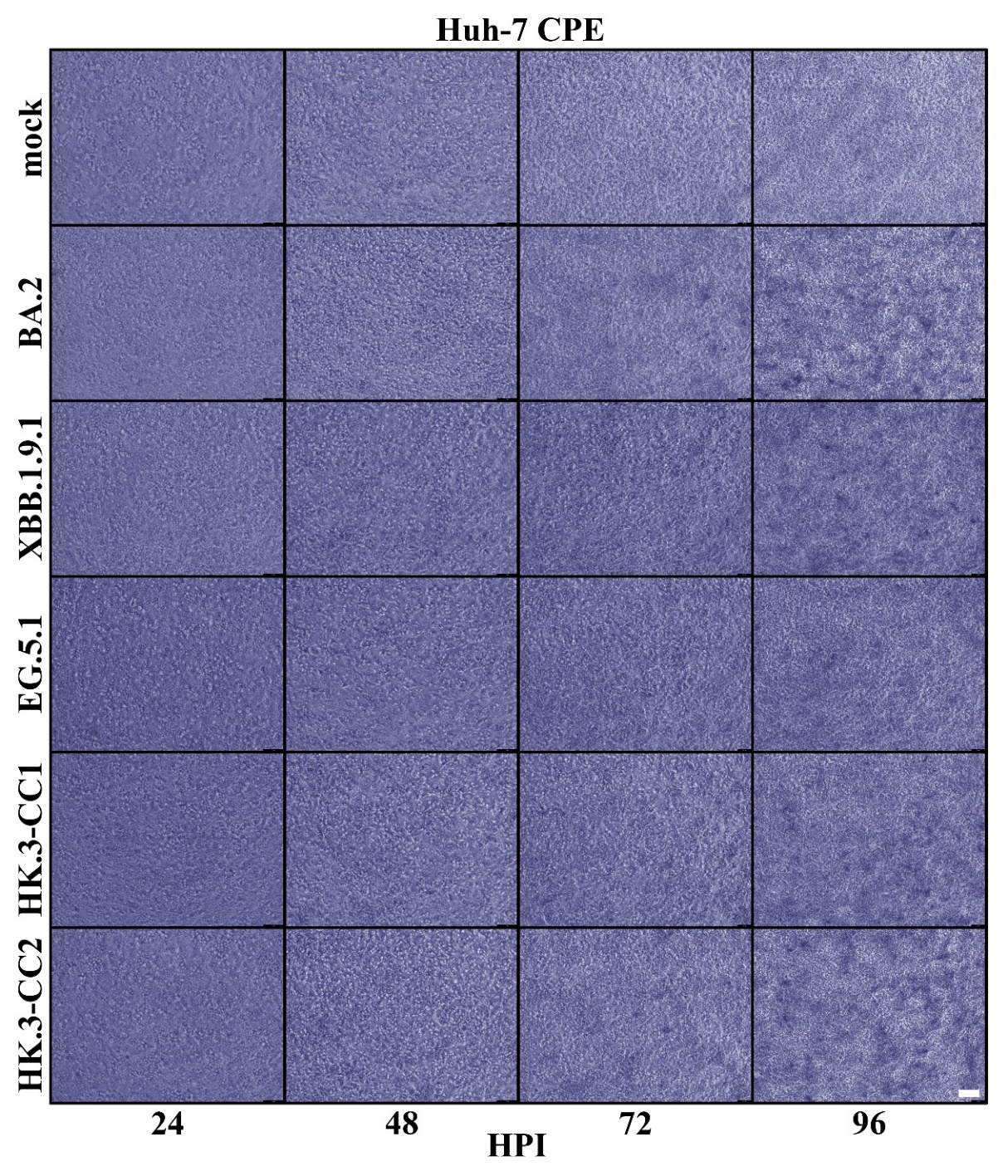


Figure S4. The image of distinct cytopathic effects for Huh-7 cells. Representative images depicting the cytopathic effects in Huh-7 following BA.2 and XBB.1.9 subvariants infection are shown from 24 to 96 HPI. Images were captured at 40× magnification; scale bar = 75 µm.


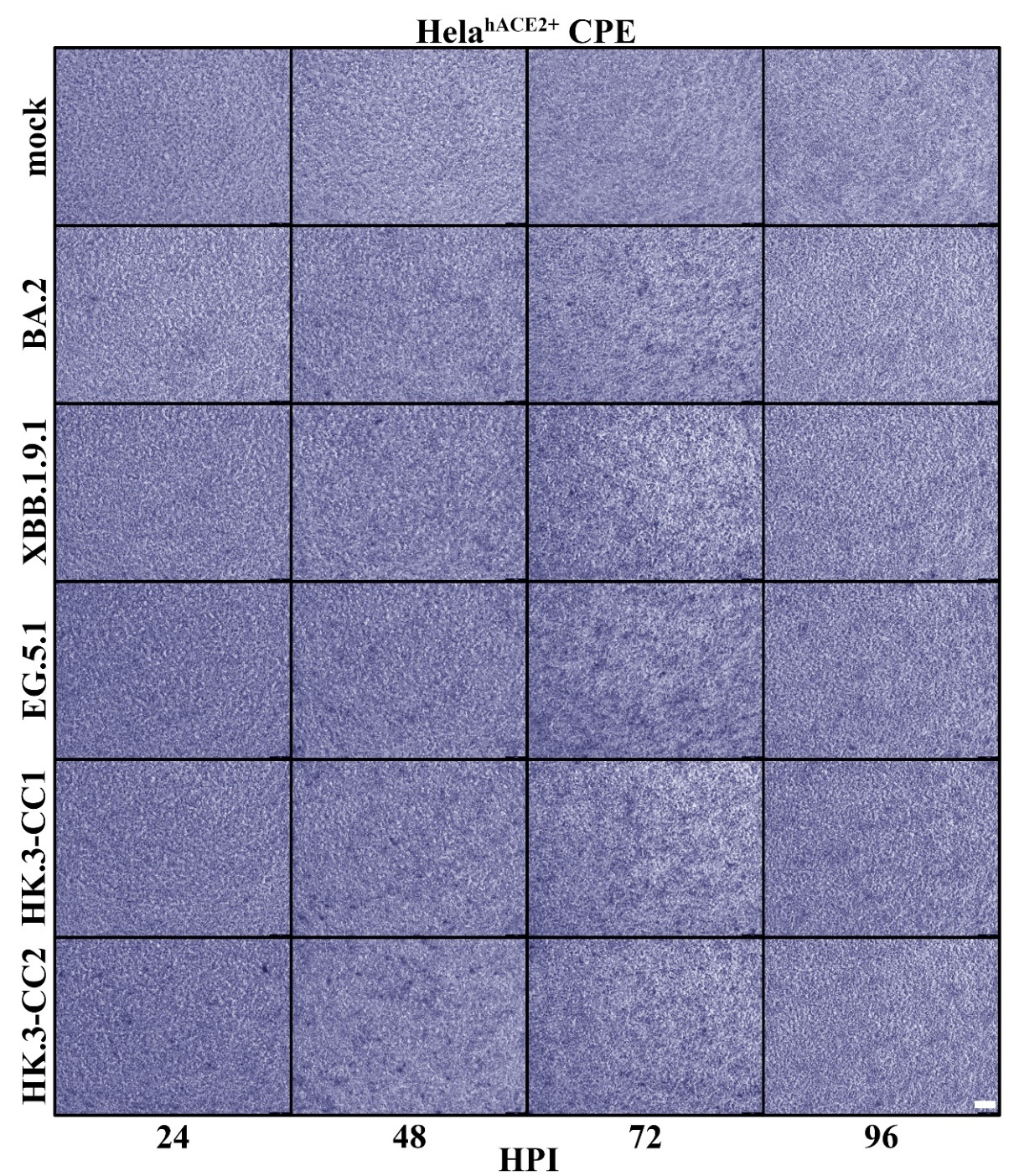


Figure S5. The image of distinct cytopathic effects for HeLa^hACE2+^ cells. Representative images depicting the cytopathic effects in HeLa^hACE2+^ following BA.2 and XBB.1.9 subvariants infection are shown from 24 to 96 HPI. Images were captured at 40× magnification; scale bar = 75 µm.


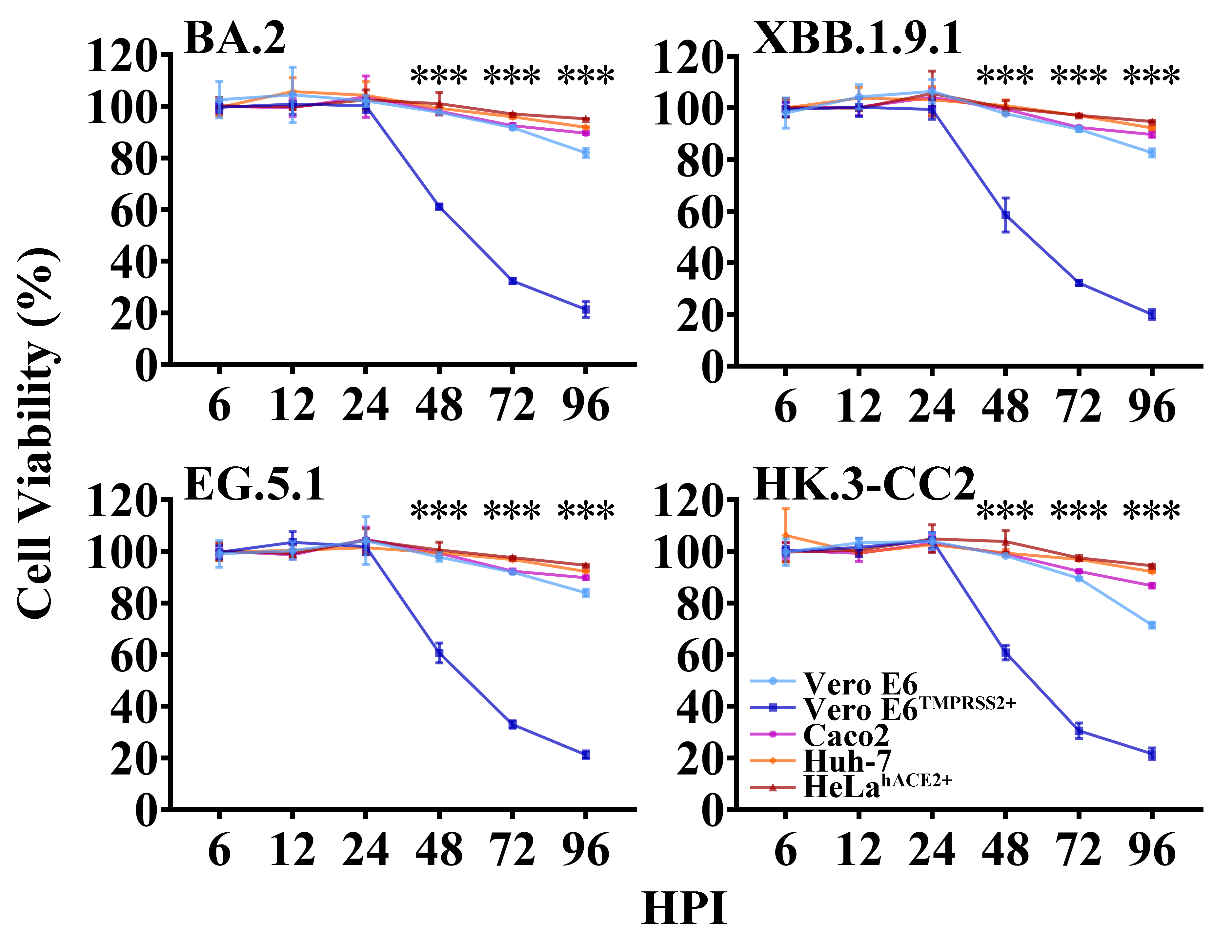


Figure S6. Viability of infected cells. Viability of Vero E6 (cyan), Vero E6^TMPRSS2+^ (blue), Caco2 (purple), Huh-7 (orange), and HeLa^hACE2+^ (dark red) cells infected with BA.2, XBB.1.9.1, EG.5.1 or HK.3-CC2. Significances of viability differences between Vero E6 and Vero E6^TMPRSS2+^ cells are revealed by black asterisks above the lines.


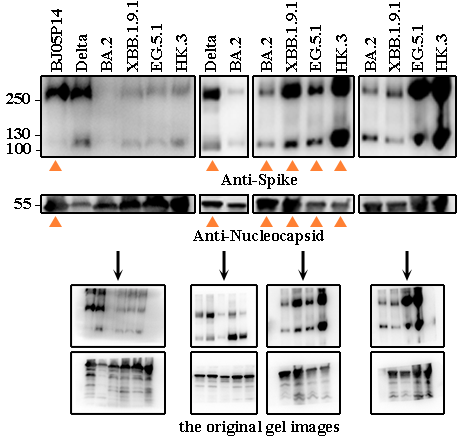


Figure S7. The original image for Figure 2D includes the complete set of three biological replicates. The lane marked with an orange triangle was selected as the representative image for presentation in the main figure.


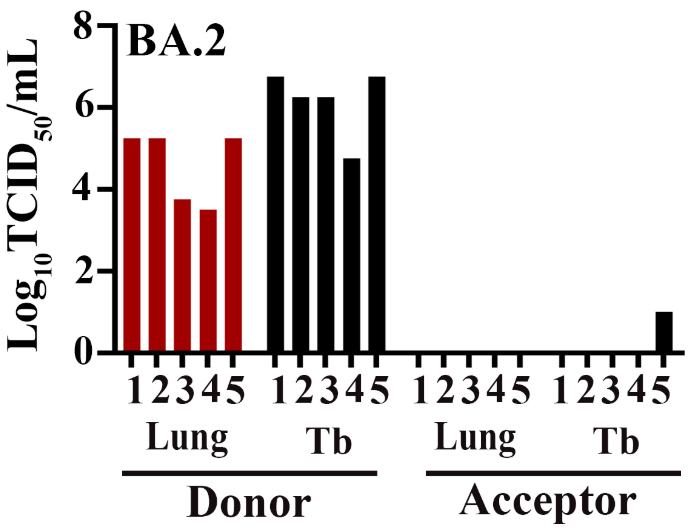


Figure S8. Airborne transmission of BA.2 in hamsters. The viral titers of the lungs and turbinates of the inoculated donors and acceptors at 5 DPI are indicated in dark red and black, respectively. These results were reproduced using identical experimental protocols as originally detailed in Figure 3A of our prior publication (*Wang W, Jin Q, Liu R, et al. Virological characteristics of SARS-CoV-2 Omicron BA.5.2.48. Front Immunol. 2024; 15:1427284. doi:10.3389/fimmu.2024.1427284*).

Table S1. Primer applications and sequences

| **Primer Name** | **Primer applications** | **Primer sequences (5’ to 3’)** |
| --- | --- | --- |
| Spike_52-F | Primer pairs 1 for in vivo competitive experiment (RT-PCR) | GGGTACTGCTGTTATGTCTTT |
| Spike_52-R |  | CTCACTTTCCATCCAACTTTT |
| Spike_456-F | Primer pairs 2 for in vivo competitive experiment (RT-PCR) | AGGTAATGAAGTCAGCCAAAT |
| Spike_456-R |  | AGCATCAGTAGTGTCAGCAAT |
| GAPDH-F | Primer pairs 3 for relative RNA expressions experiment (qRT-PCR) | aggtggtctcctctgacttc |
| GAPDH-R |  | ccaaattcgttgtcataccagg |
| TMPRSS2-F | Primer pairs 4 for relative RNA expressions experiment (qRT-PCR) | cactgcgtggaaaaacctctt |
| TMPRSS2-R |  | cagcttcatcagcgcaatgt |
| ACE2-F | Primer pairs 5 for relative RNA expressions experiment (qRT-PCR) | ttcctgctcaaacaagcactc |
| ACE2-R |  | cccaactatctctcgcttcatc |

Table S2. Antibodies and reagents information

| **Antibodies or Reagents** | **SOURCE** | **IDENTIFIER** |
| --- | --- | --- |
| **Antibodies** | | |
| anti-nucleocapsid antibody | Genetex | Cat# GTX135357; RRID: AB_2887440  (dilution, 1:1000) |
| anti-spike S2 antibody | Genetex | Cat# GTX632604; RRID: AB_2864418  (dilution, 1:1000) |
| HRP-conjugated goat-anti-rabbit antibody | ZSGB-Bio | Cat# ZB-2301  (dilution, 1:500) |
| FITC-conjugated goat-anti-rabbit antibody | Jackson ImmunoResearch | Cat# 111-095-003; RRID: AB_2337978  (dilution, 1:500) |
| **Reagents** | | |
| Dulbecco’s Modified Eagles Medium (DMEM) | Gibco (Thermo Fisher) | Cat# 11885-084 |
| Fetal Bovine Serum (FBS) | Gibco (Thermo Fisher) | Cat# A5669701 |
| 0.25% Trypsin-EDTA | Gibco (Thermo Fisher) | Cat# 25200-056 |
| Maxima H Minus cDNA Synthesis Kit | Thermo Fisher Scientific | Cat# K1652 |
| KAPA HiFi HotStart ReadyMix | Roche | Cat# KK2602 |
| QIAamp Viral RNA Mini Kit | QIAGEN | Cat# 52906 |
| iTaq Universal SYBR Green Supermix | Bio-Rad | Cat# 1725121 |
| RNAsimple Total RNA Kit | TIANGEN Biotech | Cat# DP419 |
| SuperScript IV Reverse Transcriptase | Thermo Fisher Scientific | Cat# 18091050 |
| Lipofectamine 3000 | Thermo Fisher Scientific | Cat# L3000015 |
| polyethylenimine (PEI) | Polysciences | Cat# 23966-1 |
| Cell Counting Kit-8 (CCK-8) | MedChemExpress | Cat# HY-K0301 |
| SARS-CoV-2 Nucleic Acid Test Kit | KingMed Diagnostics | Cat# JC10223-1N |
| EndoFree Plasmid Maxi Kit | QIAGEN | Cat# 12362 |
| EnduRen live cell substrate | Promega | Cat# E6481 |
| DAB substrate kit | Vector Laboratories | Cat# SK-4100 |
| Protein A sensor chip | Cytiva | Cat# 29127555 |
| HEPES running buffer | Cytiva | Cat# BR100669 |
| Strep-Tactin XT | IBA Lifesciences | Cat# 2-1202-025 |
